# Clinical Workflow in Ocular Prosthesis Fabrication: A Technique for Minimizing Patient Visits

**DOI:** 10.7759/cureus.67711

**Published:** 2024-08-25

**Authors:** Nor Aidaniza Abdul Muttlib, Rabihah Alawi

**Affiliations:** 1 Prosthodontics Unit, School of Dental Sciences, Health Campus, Universiti Sains Malaysia, Kota Bharu, MYS; 2 Conservative Dentistry Unit, School of Dental Sciences, Health Campus, Universiti Sains Malaysia, Kota Bharu, MYS

**Keywords:** ocular defect, ocular rehabilitation, eye prosthesis, maxillofacial prosthesis, ocular prosthesis

## Abstract

The traditional approach to fabricating ocular prostheses often requires multiple visits, including impression making, wax pattern try-in, iris coloring, iris button fitting, and final prosthesis delivery. This process can be challenging for patients with logistical difficulties. Additionally, not all clinics offer this service due to limitations in expertise and equipment. This case report aims to present a modified technique for creating ocular prostheses that reduces the number of clinical visits while maintaining the quality of the final result.

## Introduction

Fabrication of the ocular prosthesis is aimed at replacing the missing eye components due to various causes. Multiple case reports highlighted the fabrication of ocular prostheses, ranging from simple techniques using ready-made materials to the incorporation of digital technology [1-4]. These techniques have demonstrated advantages, such as simplifying the fabrication process and reducing chairside time. However, they also have disadvantages, including potentially less aesthetic outcomes, the need for specialized software, and higher costs. This case report introduces a modified technique designed to reduce the number of clinical visits required, combining steps typically spread across multiple appointments into fewer visits. While the lab procedures remain similar, the clinical steps are streamlined. This approach benefits patients, especially those with logistical challenges, although it necessitates longer clinical time during the initial visit without compromising the prosthesis's quality.

## Case presentation

A 66-year-old male patient was referred from the Ophthalmology Clinic at Hospital Universiti Sains Malaysia for the fabrication of a new ocular prosthesis. He recently lost his previous prosthesis while gardening. He had no known medical illness. The patient underwent evisceration of his right eye five years ago following a wood-related injury. Clinical examination revealed that the left eye socket is equal in size to the opposite eye, showed no signs of inflammation, and the orbital implant was palpable. The patient provided consent for photographs to be taken and used with appropriate documentation.

First visit

**Figure 1:**
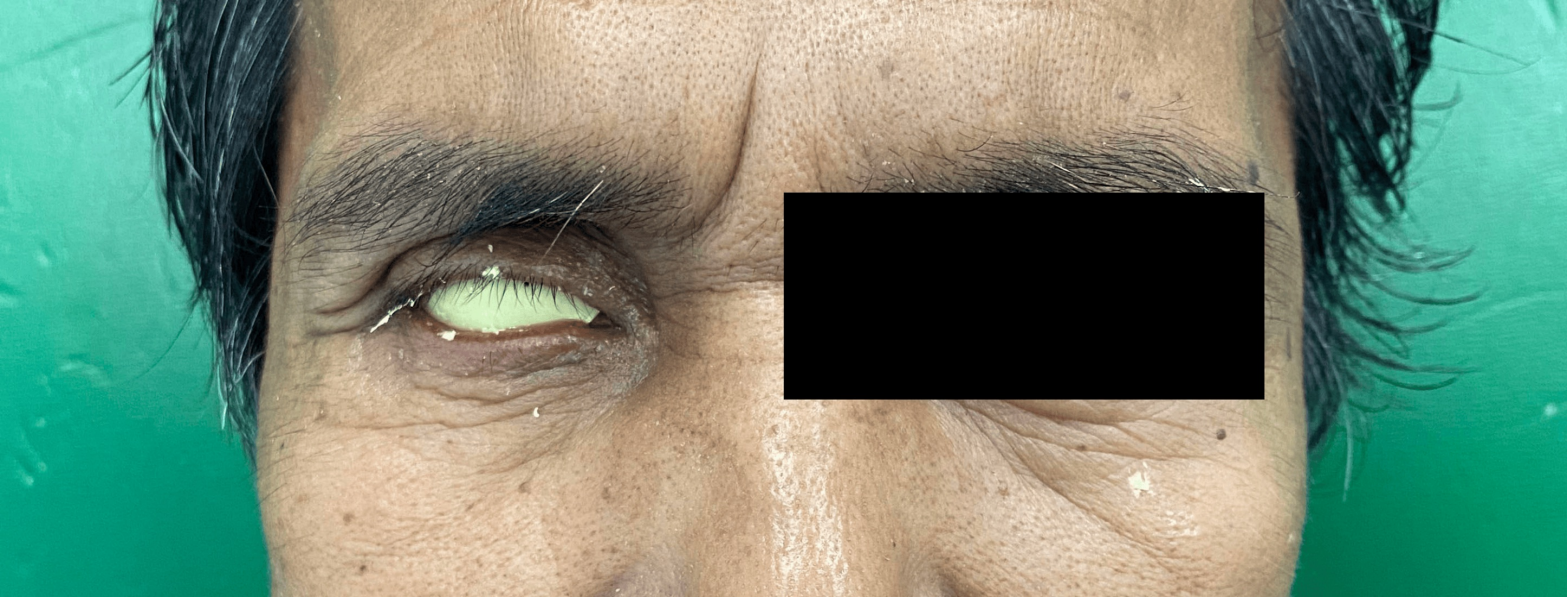
The impression inside the socket.

The impression of the socket was recorded during the first visit. The tray was selected based on the lesion's shape and size. It was then loaded with heavy-body polyvinyl siloxane (PVS) material (Examix NDS, Tokyo, Japan) and placed into the socket (Figure 1).

**Figure 2:**
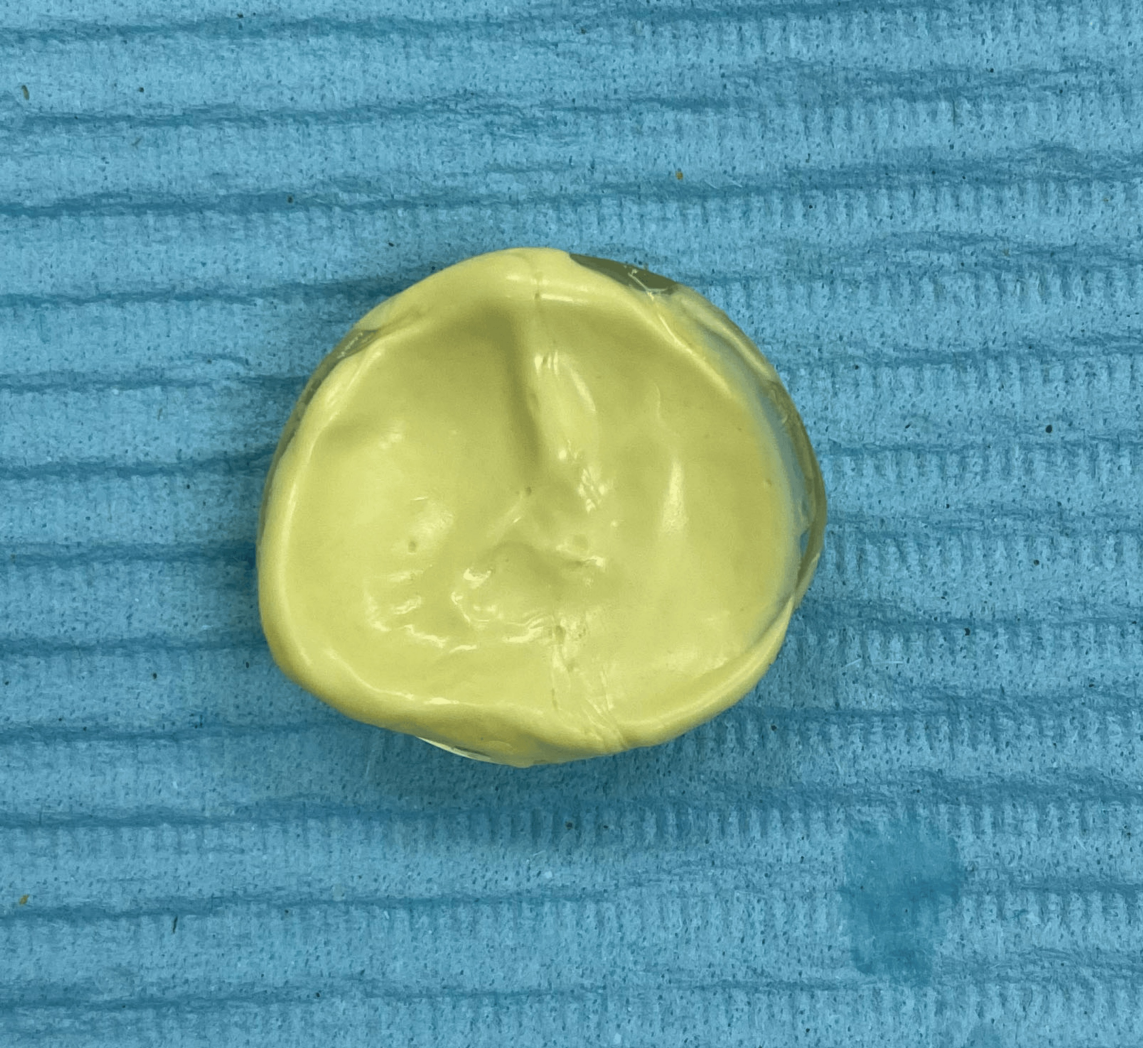
The ocular impression made from polyvinyl siloxane material.

The patient was seated upright with the head positioned straight during the impression-making process. The volume of the eye was assessed by comparing the impression side with the opposing eye from a frontal view. The patient was instructed to move their eye to the right, left, up, and down, and then relax. He was asked to stare straight ahead at a wall approximately six feet away until the material set. The impression was checked to ensure it covered up to the medial canthal area. Once the PVS had set, the impression was removed from the socket and inspected for any flaws (Figure 2).

**Figure 3:**
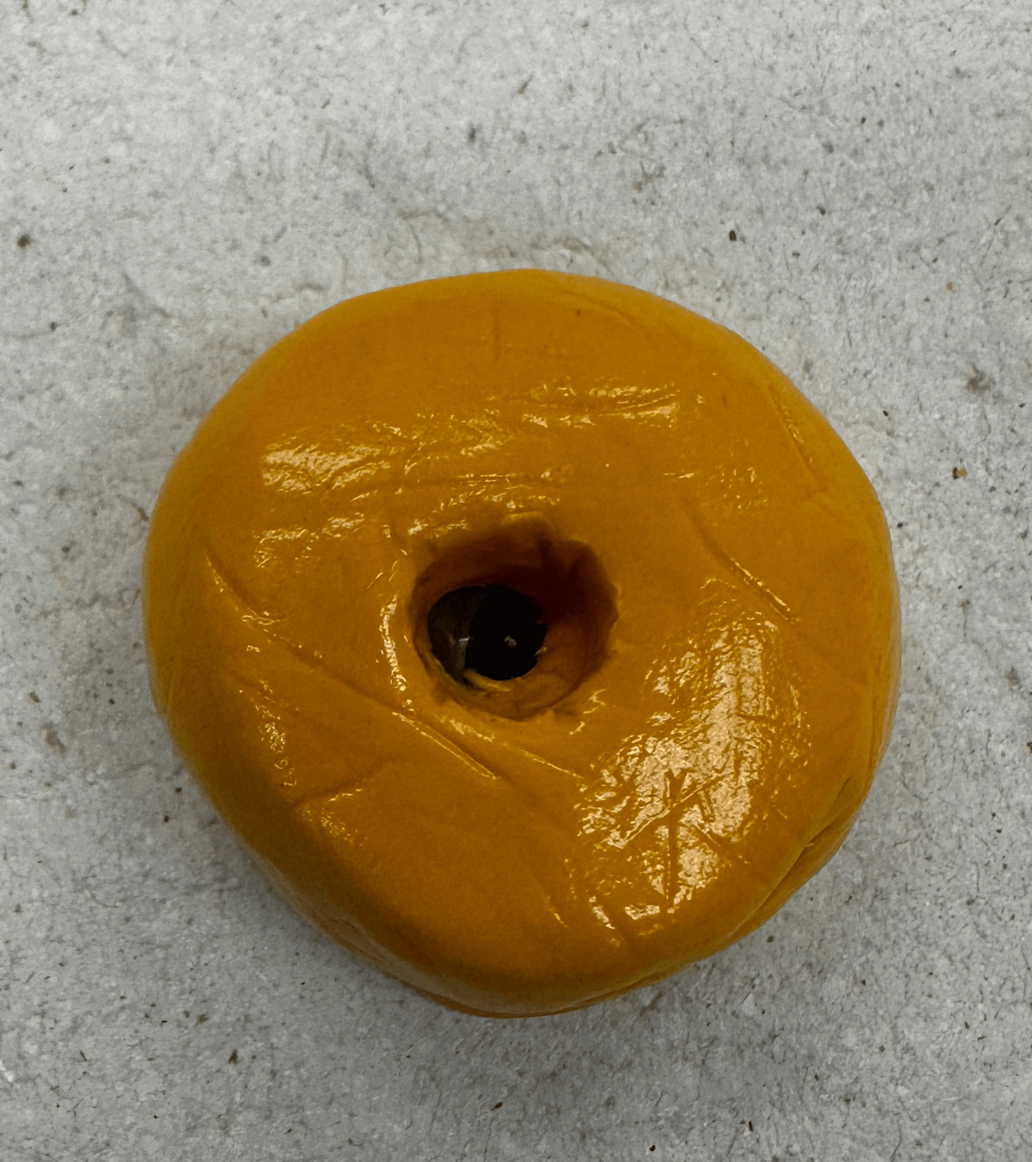
The mold for the fabrication of the wax pattern.

The mold was created using putty impression material (3M Deutschland GmbH, Neuss, Germany), as shown in Figure 3. The first part of the mold was formed by pressing the outer side of the impression onto the putty. After the putty hardened, three reference points were marked using a V notch, and Vaseline was applied to the surface. The second portion of the putty was then added on top of the exposed impression surface. A PVS impression tip was used to form the sprue channel. Once the silicone material had set, the tip was removed, the mold was separated, and the impression was taken out.

**Figure 4:**
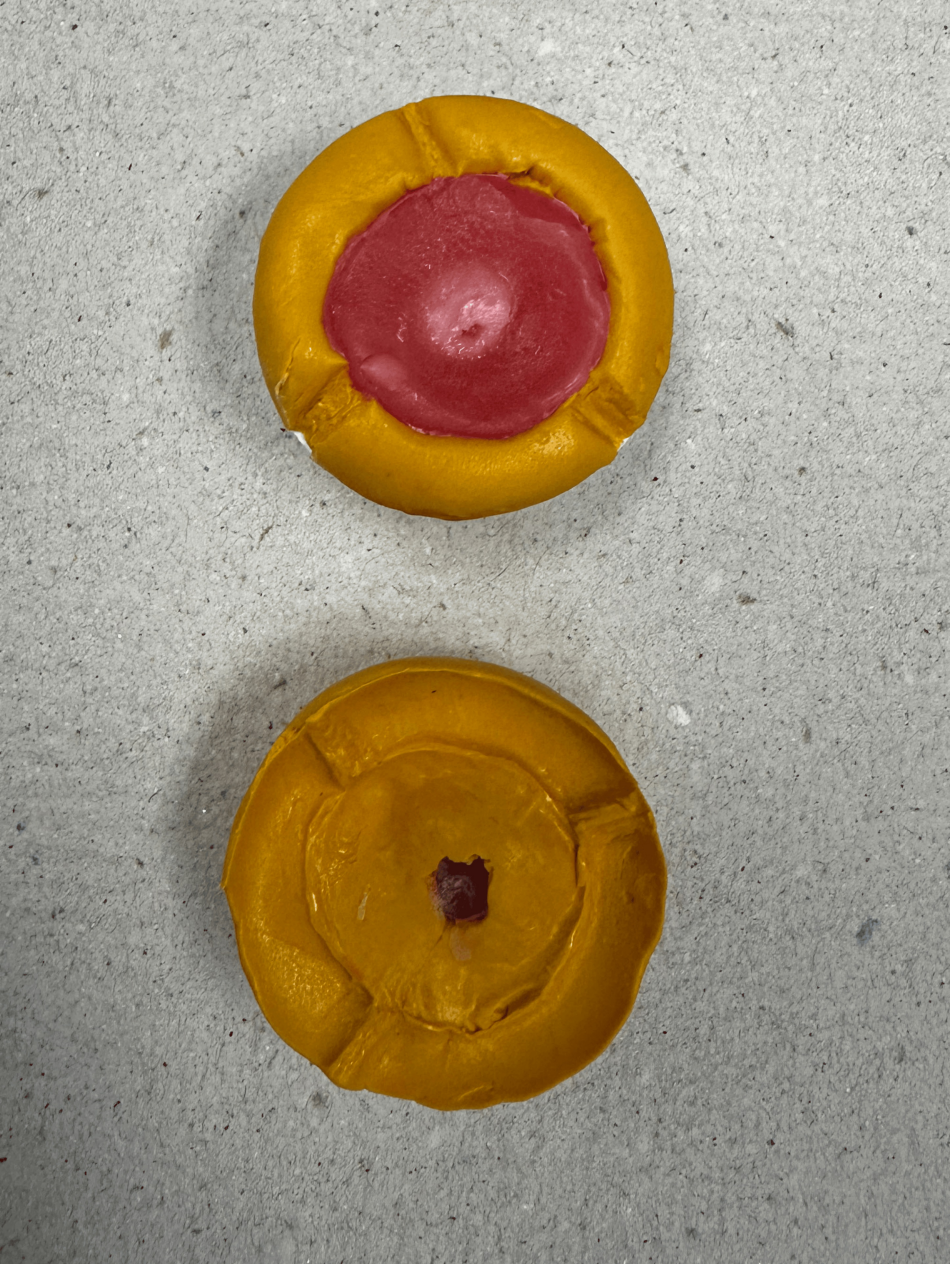
The wax pattern fabricated using the putty mold.

The mold was reassembled, and molten wax was poured into it, allowed to harden, and then removed (Figure 4). Any sharp edges were smoothed out before the try-in process, which took place during the same visit.

**Figure 5:**
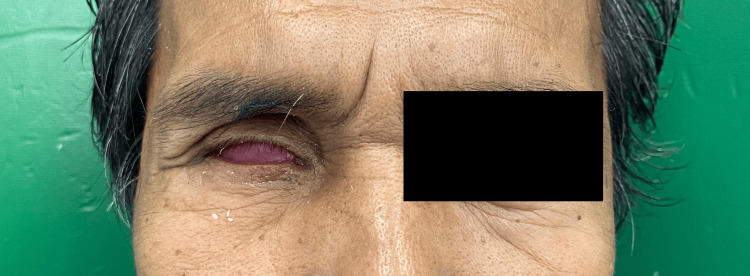
The try-in process for the wax pattern inside the socket.

The wax pattern was tried, and modifications were made to ensure the proper shape and achieve a good tissue profile (Figure 5).

**Figure 6:**
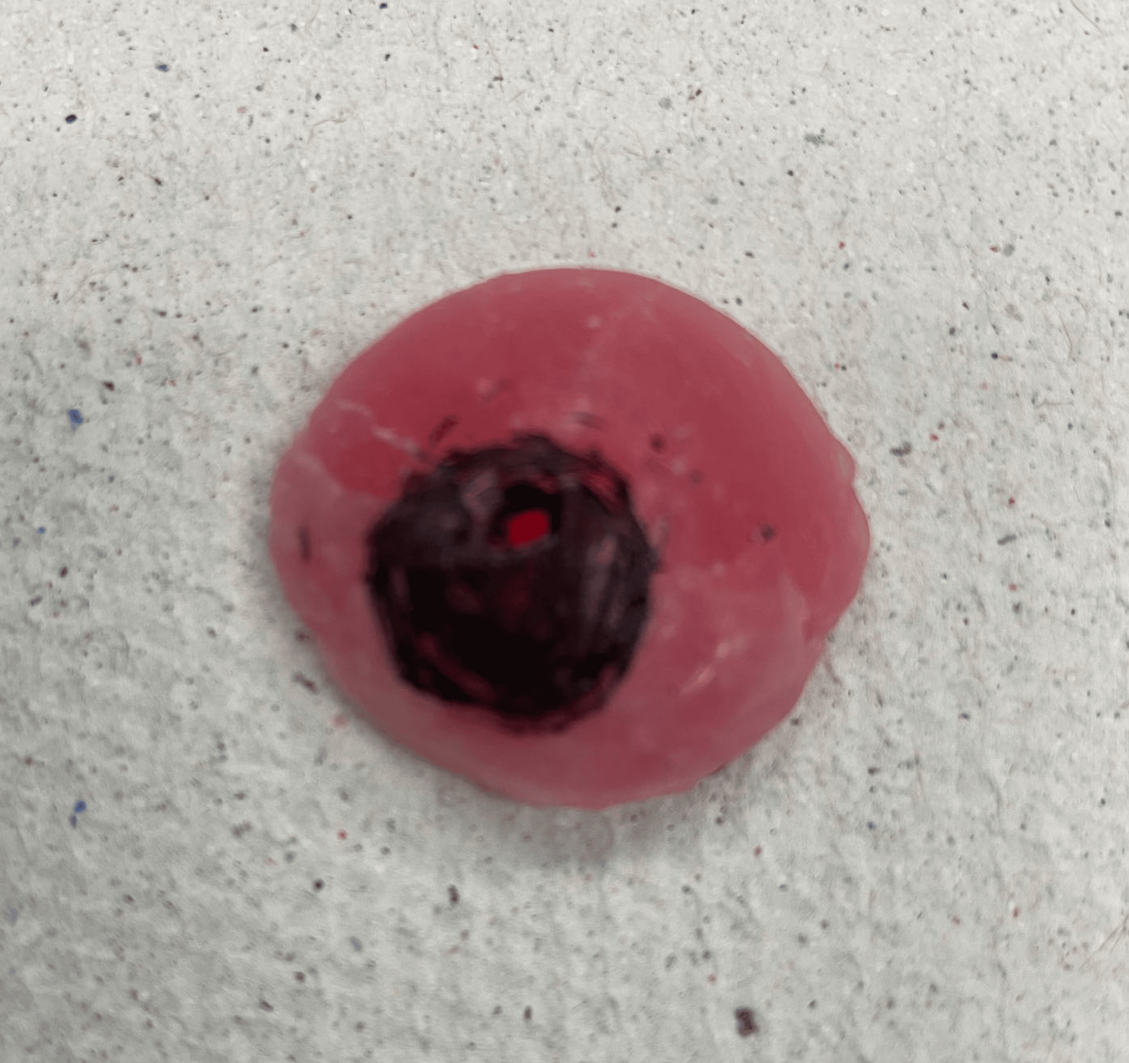
The wax pattern with iris marking.

Once the wax pattern was finalized, the iris position was marked (Figure 6), and a color-matching process was carried out. The iris and sclera shades were matched accordingly. It was recommended that a photograph of the opposing eye be taken during this visit to help the lab design the prosthesis with accurate features.

**Figure 7:**
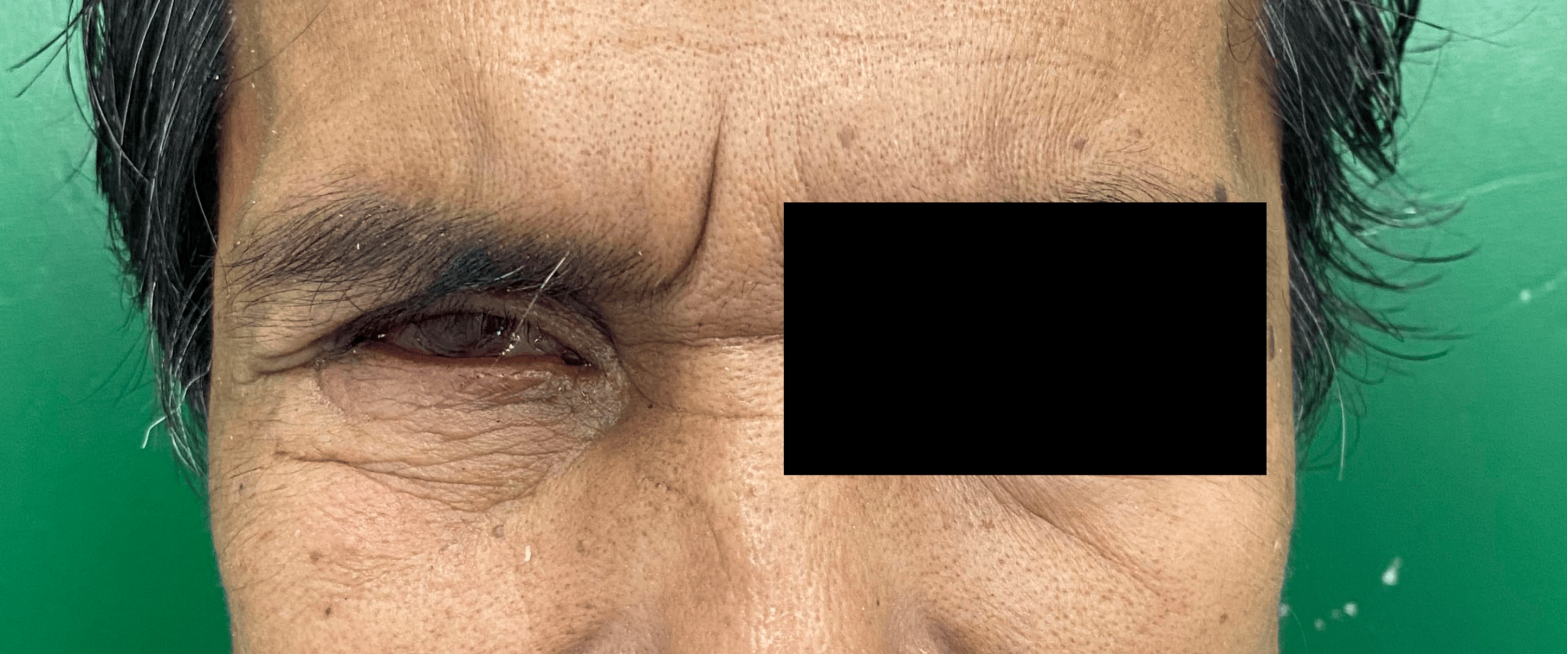
Initial conformation of the iris position inside the socket. It is very important to ensure the marking was correctly placed to guide the positioning of the iris button in the lab later.

The position and size of the iris inside the orbital cavity were confirmed (Figure 7). Iris painting was performed either chairside or later in the lab. However, it was not advised to position the iris button on the wax pattern immediately after painting to ensure the paint dried completely for at least 24 hours. The patient was discharged from the clinic following this procedure.

Second visit

**Figure 8:**
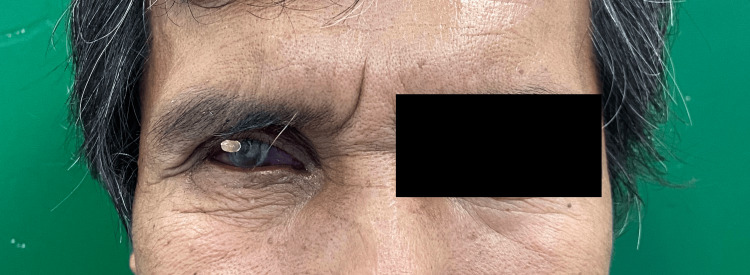
Try-in procedure with the iris button installed.

At the second appointment, the iris button was positioned and tested to ensure the correct size, shade, and placement (Figure 8). The eye's opening, volume, movements, scleral shade and characteristics, and the symmetry of the proposed prosthesis were thoroughly assessed during this visit.

Third visit

**Figure 9:**
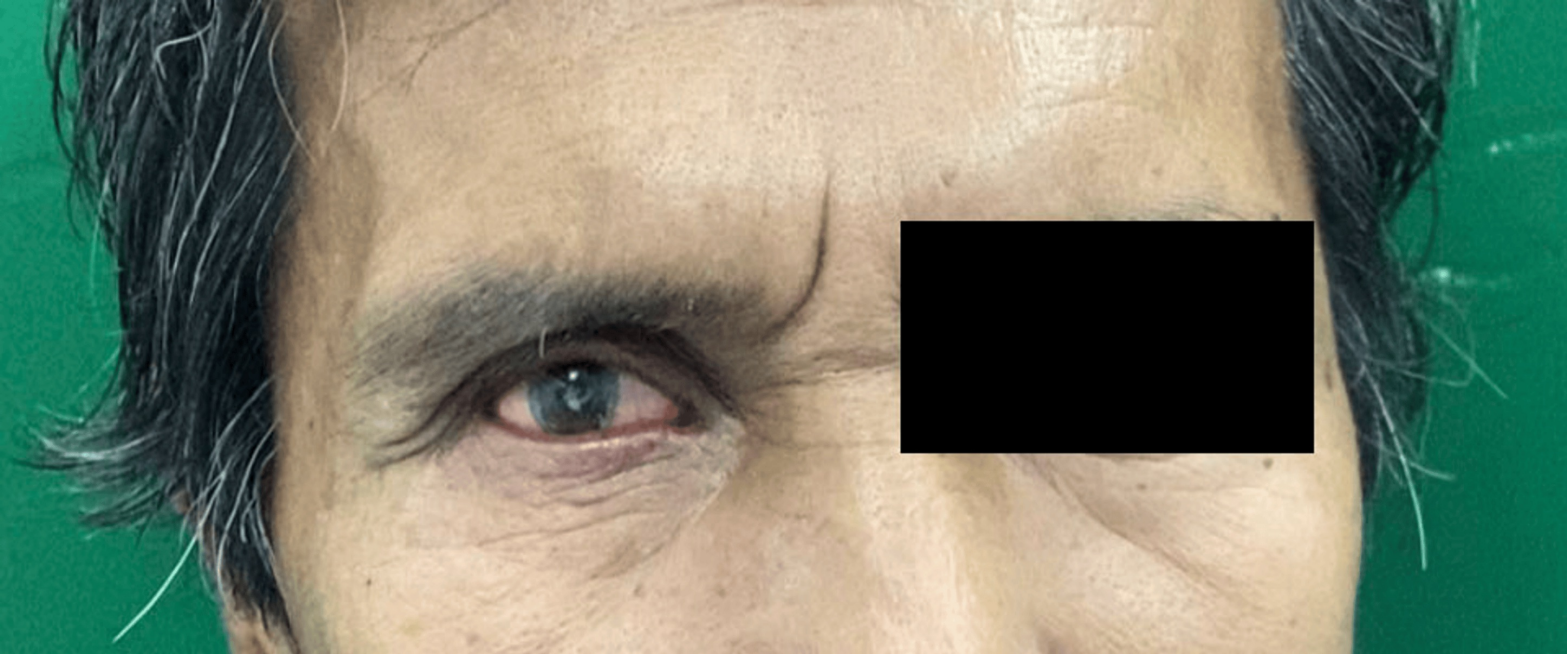
The final prosthesis.

**Figure 10:**
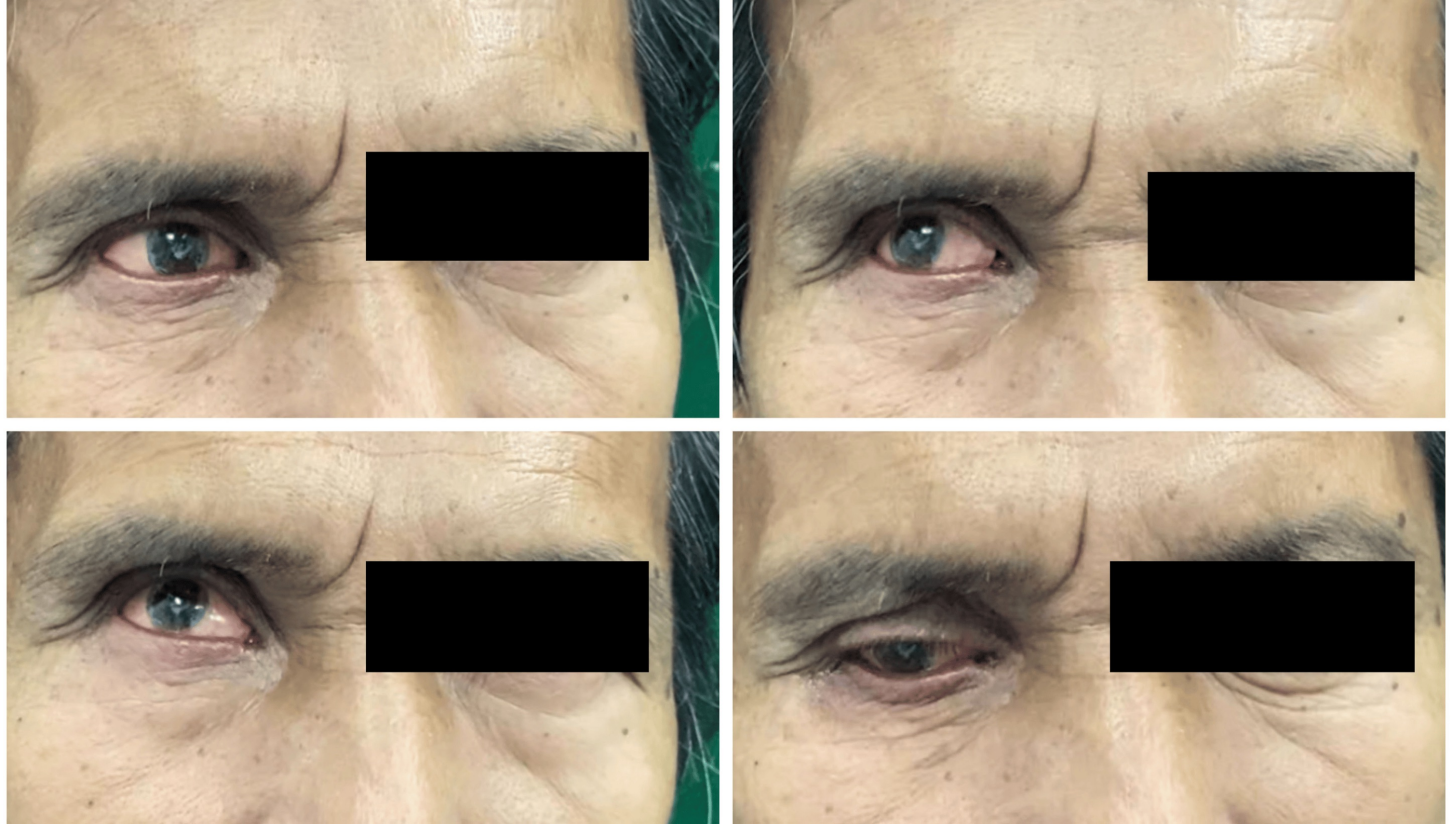
The fit and movement of the prosthesis were assessed.

At the third appointment, the ocular prosthesis was delivered, and final adjustments were made as necessary (Figure 9). The prosthesis was re-evaluated for movement (Figure 10), and approval from the patient was confirmed.

## Discussion

A proper technique in the fabrication of ocular prostheses ensures the aesthetic will be restored at best [5,6]. However, the techniques that were reported related to the fabrication of this prosthesis vary according to the center. Previous reports have shown that the fabrication of ocular prostheses involved multiple clinical visits and several laboratory stages [2]. It was noted that the process often required separate clinical sessions for wax pattern adjustments and try-ins, resulting in an increased number of patient appointments. Our case report presents a method designed to reduce the number of required visits. This technique consolidates procedures, with lab stages focused solely on processing the prosthesis. Fewer visits benefit patients, particularly those with logistical challenges. However, this approach requires a longer initial clinical visit to complete the wax pattern, iris positioning, and color matching on the same day. Iris painting is done independently to save chairside time and allow for detailed work. A high-quality photograph of the opposing eye is crucial for accurate prosthesis design. This method contrasts with conventional techniques, where impressions are sent to the lab, and a study model is created from dental stone before wax pattern fabrication and adjustment in subsequent visits [2].

With our technique, the first visit is longer, and iris positioning and try-in are performed in a second visit to ensure proper drying of the paint. Alternatively, iris positioning and try-in could be done on the same visit using a prefabricated iris button, though this may compromise outcomes due to potential mismatches in iris shade and character. This method is more challenging for lighter-colored irises, particularly in Caucasian patients. Additional advantages include reduced involvement of dental technicians and the ability of the operator to control and adjust the wax pattern details during the same visit.

## Conclusions

The fabrication of ocular prostheses can vary depending on the center. To address patient needs and challenges, it is possible to combine several procedures into a single visit without compromising the final outcome of the prosthesis. This approach benefits the patient by reducing the number of clinic visits, though it does require more chairside time for the clinician. Further evaluation is needed to assess the longevity and quality of prostheses produced using this technique.
